# Draft genome sequence, annotation and SSR mining data of *Oryctes rhinoceros* Linn. (Coleoptera: Scarabaeidae), the coconut rhinoceros beetle

**DOI:** 10.1016/j.dib.2021.107424

**Published:** 2021-09-29

**Authors:** Rajesh M. K, Ginny Antony, Kumar Arvind, Jeffrey Godwin, Gangaraj K. P, Sujithra M, Josephrajkumar A, Tony Grace

**Affiliations:** a ICAR-Central Plantation Crops Research Institute, Kasaragod, Kerala 671124, India; b Central University of Kerala, Kasaragod, Kerala 671320, India; c Bionivid Technology Private Limited, Bengaluru, Karnataka 560043, India; d Regional Station, ICAR-Central Plantation Crops Research Institute, Kayamkulam 690533, India

**Keywords:** Coconut, Rhinoceros beetle, Whole-genome sequencing, Genomics, SSRs

## Abstract

The coconut rhinoceros beetle (CRB), *Oryctes rhinoceros* Linn. (Coleoptera: Scarabaeidae), is one of the major pests of coconut causing severe yield losses. The adult beetles feed on unopened spear leaf (resulting in the typical ‘V’-shaped cuts), spathes, inflorescence, and tender nut leading to stunted palm growth and yield reduction. Moreover, these damages serve as predisposing factors to the entry of other fatal enemies on palms, *viz.,* red palm weevil and bud rot disease, causing yield loss as high as 10%. CRB attacks juvenile palms through the collar region, affecting the growth and initial establishment of the juvenile palms. While the immature stages of CRB sustain on organic debris, the adult beetles are ubiquitous pests on coconut and other palms. The discovery of a new invasive haplotype of CRB from Guam and other Pacific Islands, insensitive to *Oryctes rhinoceros* nudivirus (*OrNV*), a potent biocontrol agent, has raised serious concerns. The draft genome sequence and simple sequence repeat (SSR) marker data for this important pest of coconut are presented here. A total of 30 Gb of sequence data from an individual third instar larva was obtained on an Illumina HiSeq X Five platform. The draft genome assembly was found to be 372 Mb, with 97.6% completeness based on Benchmarking Universal Single-Copy Orthologs (BUSCO) assessment. Functional gene annotation predicted about 16,241 genes. In addition, a total of 21,999 putative simple sequence repeat (SSR) markers were identified. The obtained draft genome is a valuable resource for comprehending population genetics, dispersal patterns, phylogenetics, and species behavior.

## Specifications Table

**Table:** 

Subject	Agriculture sciences
Specific subject area	Insect genomics
Type of data	Tables, figures, text files
How data were acquired	Illumina HiSeq X Five sequencing platform
Data format	Raw, filtered, analyzed
Parameters for data collection	DNA from the whole body of one individual third instar larva (60 days) was used
Description of data collection	DNA from the whole body of one individual third instar larva (60 days) was extracted. DNA purity and concentration were assessed prior to sequencing. The sequencing was undertaken on the Illumina HiSeq X Five platform, followed by *de novo* assembly using ABySS.
Data source location	Kasaragod, India (12°32′38.0″N; 74°57′45.7″E).
Data accessibility	Repository name: NCBI SRA Data identification number: PRJNA724335 Assembly: NCBI Accession no. JAHRIJ000000000 Direct URL to data: https://www.ncbi.nlm.nih.gov/sra/SRX10668900

## Value of the Data

• The dataset provides the draft genome sequence for a notorious pest of coconut, the coconut rhinoceros beetle (CRB) (*Oryctes rhinoceros*).
• This dataset will be useful to palm entomologists working in the area of genomics and phylogenetics.
• The dataset would enable the prediction of genes conferring pesticide resistance in this economically important coleopteran.
• The genome can be mined to identify effective and novel targets for the control of CRB and targeting of specific genes with molecular tools like RNAi (RNA interference).

## 1 Data Description

The first draft genome assembly, annotation, and SSR marker data of the *Oryctes rhinoceros* Linn. (Coleoptera: Scarabaeidae) is presented in this article. A total of 215 million reads (equating to 30 Gb) data was generated after sequencing. A summary of statistics on the draft genome and its features are provided in Table 1. The assembly consisted of 25,242 scaffolds with an N50 of 5.42 Mb (Table 1) and was 372.39 Mb in total length. The 355.25 Mb genome size estimated with *k-*mer spectra using Jellyfish (with *k*-mer size set as 77) is complementary to these estimates. Table 2 provides the assessment of genome completeness using the BUSCO tool. The analysis revealed 96.9% of the core Insecta orthologs were complete and single copy, 0.7% complete and duplicated, 1.4% fragmented, and 1% missing. These results indicate that the genome is of good quality.

**Table 1:** Draft genome assembly statistics of *Oryctes rhinoceros*.

	Contig- ABySS (*k* = 77)	Scaffold- BESST	RaGOO (with GCA_902654985.1)	RaGOO (>= 1Kb)
Total sequences	1053,350	913,021	877,751	25,242
Total bases	536,020,998	526,785,513	530,312,513	372,388,193
Min sequence length	77	77	77	1000
Max sequence length	428,931	837,675	19,770,802	19,770,802
Average sequence length	508.87	576.97	604.17	14,752.72
Median sequence length	151	153	153	1799
N25 length	8648	14,027	7578,861	9393,814
N50 length	3440	5624	2162,532	5428,920
N75 length	441	526	536	888,259
N90 length	153	153	154	3530
N95 length	91	108	108	1900
As	31.05%	30.99%	30.77%	31.53%
Ts	31.02%	30.96%	30.77%	31.57%
Gs	18.97%	18.89%	18.76%	17.78%
Cs	18.95%	18.88%	18.76%	17.77%
(*A* + *T*)s	62.08%	61.95%	61.54%	63.11%
(*G* + *C*)s	37.92%	37.77%	37.52%	35.55%
Ns	0.00%	0.28%	0.94%	1.34%

**Table 2:** Assessment of genome completeness of *Oryctes rhinoceros* using BUSCO.

Insecta_odb10	Num	%
Complete BUSCOs (C)	1334	97.6
Complete and single-copy BUSCOs (S)	1325	96.9
Complete and duplicated BUSCOs (D)	9	0.7
Fragmented BUSCOs (F)	19	1.4
Missing BUSCOs (M)	14	1
Total BUSCO groups searched	1367	

**Fig. 1:**
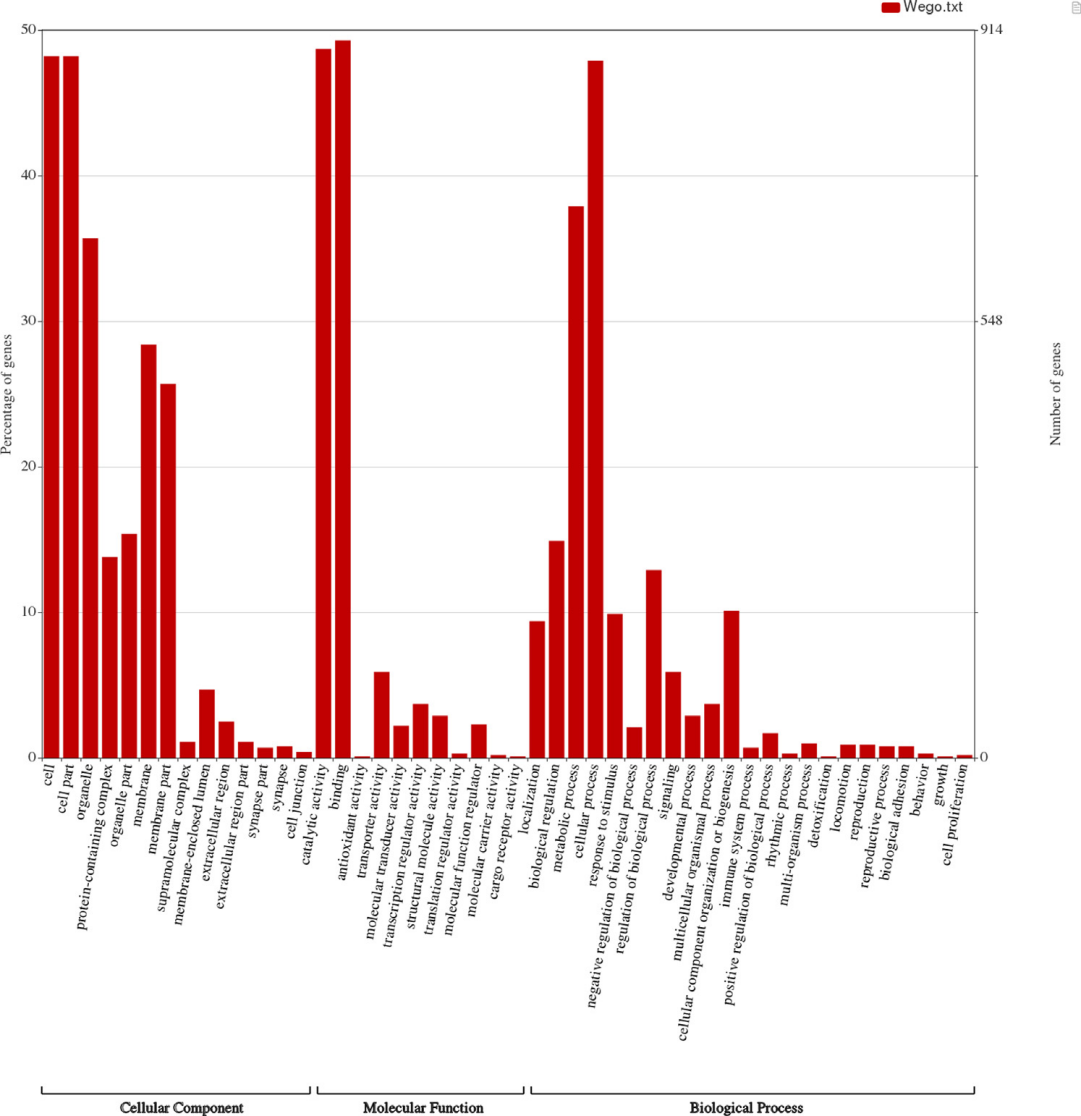
Histogram representing the gene ontology distribution of the annotated *Oryctes rhinoceros* genes. The functionally annotated genes were assigned to three main GO categories: Cellular Component, Molecular Function, and Biological Process.

Detailed information of the repetitive elements detected in the assembled CRB genome is provided in Supplementary file S1. About 90.3 Mb (24.2%) of repeats were predicted in the draft genome of CRB. Functional gene annotation pipeline predicted about 16,241 genes, of which 13,779 gene isoforms were annotated with NCBI RefSeq and UniProt databases. The GO term classification and distribution, visualization using Web Gene Ontology Annotation Plot (WEGO), can be seen in Fig. 1. The assembled draft genome of CRB was also used for the identification of simple sequence repeat (SSR) or microsatellite markers, the details of which are provided in Table 3. Totally, 21,999 SSRs were identified, of which 1414 SSRs were present in compound formation.

**Table 3:** Statistics of SSRs predicted from *Oryctes rhinoceros* genome.

Total number of sequences examined	25,242
Total size of examined sequences (bp)	372,388,193
Total number of identified SSRs	21,999
Number of SSR containing sequences	2334
Number of sequences containing more than 1 SSR	491
Number of SSRs present in compound formation	1414

Repeat types	Number

Mono	1358
Di	10,361
Tri	8635
Tetra	1367
Penta	217
Hexa	61

## 2 Experimental Design, Materials and Methods

### 2.1 DNA extraction and sequencing

A standardized procedure was used for the rearing of the beetle. One individual third instar larva (60 days) was taken, and DNA was extracted from the whole body using DNeasy Blood & Tissue Kit (Qiagen). The sample DNA concentration was ascertained using a Qubit 4 Fluorometer (Thermo Fisher Scientific). The DNA library was prepared using KAPA HyperPlus Kit (Roche) as per standard protocol. Sequencing was undertaken on an Illumina Hiseq X Five platform.

### 2.2 Data pre-processing and genome profiling

The raw sequence reads were initially processed by fastp (v0.2.0) [1] to trim adapter sequences and eliminate low-quality reads. The *k*-mer profile was then computed, with the values of *k* ranging between 71 and 101, with an interval of 4, using Jellyfish (v2.3.0) [2]. The obtained *k*-mer frequencies were processed to estimate major genome characteristics, including genome size, heterozygosity and repetitiveness employing GenomeScope [3]. A *k*-mer size of 77 was recommended by KmerGenie (v1.7051) [4] for an optimal genome assembly.

### 2.3 Genome assembly and evaluation

The primary assembly was constructed using ABySS 2.0 [5]. BESST [6] was used for performing scaffolding on the primary assembly. The assembly was further polished by reference-based scaffolding. RaGOO [7] was used with the available *Oryctes borbonicus* genome [NCBI Accession No. GCA_902654985.1] to reorient and improve the assembly. All contigs below 1000 bp were discarded in the final assembly. Finally, the assembled genome was evaluated for completeness with BUSCO [8] by searching against the insecta_odb10 database.

### 2.4 Gene prediction and annotation

Repeats prediction was made using RepeatMasker [9] by combining repeat librarians from LTRdigest [10], TransposonPSI [11] and RepeatModeler [12]. Gene prediction was made using the MAKER [13] pipeline incorporating Genemark-ES [14] and Augustus [15] and using transcriptome and proteins of related species as evidence. NCBI Blastx (v2.11) [16] was leveraged for the annotation of predicted genes using RefSeq [17] and UniProt [18] protein databases. They were classified into Gene Ontology categories and visualized using Web Gene Ontology Annotation Plot (WEGO) 2.0 [19].

### 2.5 Identification of simple sequence repeats (SSRs)

Using MISA-web [20], with the parameters of '10′ for mono, '6′ for di, and '5′ for tri-, tetra-, penta- and hexa- nucleotide motifs, all assembled contigs of CRB were screened for the existence of simple sequence repeats (SSRs).

## Ethics Statement

Not applicable.

## CRediT authorship contribution statement

**Rajesh M. K:** Conceptualization, Supervision, Funding acquisition, Resources, Writing – original draft, Data curation, Formal analysis. **Ginny Antony:** Conceptualization, Supervision, Funding acquisition, Resources, Writing – review & editing. **Kumar Arvind:** Writing – review & editing, Data curation, Formal analysis. **Jeffrey Godwin:** Writing – original draft, Data curation, Formal analysis. **Gangaraj K. P:** Data curation, Formal analysis. **Sujithra M:** Methodology, Writing – review & editing. **Josephrajkumar A:** Methodology, Writing – review & editing. **Tony Grace:** Conceptualization, Supervision, Funding acquisition, Resources, Writing – review & editing, Data curation, Formal analysis.

## Declaration of Competing Interest

The authors declare that they have no known competing financial interests or personal relationships that could have appeared to influence the work reported in this paper.

## Appendix Supplementary materials

S1: Summary of repetitive elements in the assembled genome of *Oryctes rhinoceros.* Image, application 1

## References

[1] Chen S., Zhou Y., Chen Y., Gu J. (2018). fastp: an ultra-fast all-in-one FASTQ preprocessor. Bioinformatics.

[2] Marçais G., Kingsford C. (2011). A fast, lock-free approach for efficient parallel counting of occurrences of *k*-mers. Bioinformatics.

[3] Vurture G.W., Sedlazeck F.J., Nattestad M., Underwood C.J., Fang H., Gurtowski J., Schatz M.C. (2017). GenomeScope: fast reference-free genome profiling from short reads. Bioinformatics.

[4] Chikhi R., Medvedev P. (2014). Informed and automated *k-*mer size selection for genome assembly. Bioinformatics.

[5] Jackman S.D., Vandervalk B.P., Mohamadi H., Chu J., Yeo S., Hammond S.A., Jahesh G., Khan H., Coombe L., Warren R.L., Birol I. (2017). ABySS 2.0: resource-efficient assembly of large genomes using a Bloom filter. Genome Res..

[6] Sahlin K., Vezzi F., Nystedt B., Lundeberg J., Arvestad L. (2014). BESST-Efficient scaffolding of large fragmented assemblies. BMC Bioinformatics.

[7] Alonge M., Soyk S., Ramakrishnan S., Wang X., Goodwin S., Sedlazeck F.J., Lippman Z.B., Schatz M.C. (2019). RaGOO: fast and accurate reference-guided scaffolding of draft genomes. Genome Biol..

[8] Simão F.A., Waterhouse R.M., Ioannidis P., Kriventseva E.V., Zdobnov E.M. (2015). BUSCO: assessing genome assembly and annotation completeness with single-copy orthologs. Bioinformatics.

[9] A.F.A. Smit, R. Hubley, P. Green, RepeatMasker (http://repeatmasker.org). Assessed 15 March 2021.

[10] Steinbiss S., Willhoeft U., Gremme G., Kurtz S. (2009). Fine-grained annotation and classification of de novo predicted LTR retrotransposons. Nucleic Acids Res..

[11] B.J. Haas, TransposonPSI. http://transposonpsi.sourceforge.net. Accessed 16 March 2021.

[12] A. Smit, R. Hubley, RepeatModeler open-1.0. http://www.repeatmasker.org. Accessed 16 March 2021.

[13] Holt C., Yandell M. (2011). MAKER2: an annotation pipeline and genome-database management tool for second-generation genome projects. BMC Bioinform..

[14] Lomsadze A., Burns P.D., Borodovsky M. (2014). Integration of mapped RNA-Seq reads into automatic training of eukaryotic gene finding algorithm. Nucleic Acids Res..

[15] Stanke M., Steinkamp R., Waack S., Morgenstern B. (2004). AUGUSTUS: a web server for gene finding in eukaryotes. Nucleic Acids Res..

[16] Camacho C., Coulouris G., Avagyan V., Ma N., Papadopoulos J., Bealer K., Madden T.L. (2009). BLAST+: architecture and applications. BMC Bioinform..

[17] O'Leary N.A., Wright M.W., Brister J.R., Ciufo S., Haddad D., McVeigh R., Rajput B., Robbertse B., Smith-White B., Ako-Adjei D., Astashyn A. (2016). Reference sequence (RefSeq) database at NCBI: current status, taxonomic expansion, and functional annotation. Nucleic Acids Res..

[18] Consortium UniProt (2015). UniProt: a hub for protein information. Nucleic Acids Res..

[19] Ye J., Zhang Y., Cui H., Liu J., Wu Y., Cheng Y., Xu H., Huang X., Li S., Zhou A., Zhang X. (2018). WEGO 2.0: a web tool for analyzing and plotting GO annotations, 2018 update. Nucleic Acids Res..

[20] Beier S., Thiel T., Münch T., Scholz U., Mascher M. (2017). MISA-web: a web server for microsatellite prediction. Bioinformatics.

